# Improving the treatment of pre-operative anemia in hepato-pancreato-biliary patients: a quality improvement initiative

**DOI:** 10.1186/s13037-020-00239-5

**Published:** 2020-04-24

**Authors:** Terry M. Zwiep, Richard W. D. Gilbert, Husein Moloo, Donna Touchie, Guillaume Martel, Tom Wallace, Kimberly A. Bertens

**Affiliations:** 1 Department of Surgery, London Health Sciences Centre, London, Canada; 2 Department of Surgery, The Ottawa Hospital – General Campus, 501 Smyth Road, Ottawa, ON K1H 8 L6 Canada; 3 Surgical Blood Management Program, The Ottawa Hospital, Ottawa, Canada

**Keywords:** Pre-operative anemia, HPB, Surgery, Quality improvement

## Abstract

**Background:**

Pre-operative anemia is a common, but treatable, condition encountered by surgical patients. It has been associated with increased perioperative complications, length of stay, and blood transfusions. The aim of this project was to increase the treatment rate of pre-operative anemia to 75% of patients consented for major hepato-pancreato-biliary (HPB) surgery.

**Methods:**

This was an interrupted time series study and a spread initiative from a similar project in a colorectal surgery population. Interventions included an anemia screening and treatment algorithm, standardized blood work, referral to a patient blood management program, and standardized oral iron prescriptions. The primary outcome measure was the change in pre-operative anemia treatment rate and the secondary outcome measure was the post treatment increase in hemoglobin.

**Results:**

A total of 208 patients were included (*n* = 124 pre-intervention and *n* = 84 post-intervention). Anemia was present in 39.9% of patients. The treatment rate of pre-operative anemia increased to 44.1% from 28.6%. The mean hemoglobin increased from 110 g/L to 119 g/L in patients who were treated (*p* = 0.03). There was no significant increase or decrease in blood transfusions or mean number of red cell units transfused per patient. Screening rates for pre-operative anemia increased from 41.1 to 64.3% and appropriate referrals to the patient blood management program increased from 14.3 to 67.6%.

**Conclusions:**

This study demonstrates a small scale spread initiative focused on the treatment of pre-operative anemia. Although the goal to treat 75% of anemic patients was not reached, an effective referral pathway to an existing patient blood management program was developed, and a significant increase in the mean hemoglobin in anemic patients who have been treated pre-operatively was demonstrated.

## Background

Anemia is defined as low blood hemoglobin concentration, and affects approximately 24.8% of the global population based on current World Health Organization definitions for anemia (< 120 g/L for women and < 130 g/L for men) [1, 2]. The prevalence of pre-operative anemia in patients undergoing all types of major surgery is even higher than the general population at 35% [3]. In hepato-pancreato-biliary (HPB) surgery patients, the rate of pre-operative anemia has been estimated to be 16.9–32.8%, and has been associated with increased perioperative complications including morbidity, length of stay, and requirement for a perioperative blood transfusion [4–10]. Additionally, a number of studies have identified an association of perioperative blood transfusions with worse long-term overall and disease-free survival in cancer patients [11–14]. Misconceptions about the prevalence, impact, and treatment strategies for pre-operative anemia lead to low treatment rates [15]. However, effective strategies such as patient blood management programs and treatment algorithms have been developed and should be utilized to address pre-operative anemia [16–18].

Patient blood management programs have been established in some institutions to improve patient outcomes by identifying and treating pre-operative anemia, addressing perioperative coagulopathy, minimizing blood loss during surgery, and using an individualized approach to each patient [19]. Identifying and treating pre-operative anemia is necessary as the aim is optimization prior to surgery. Frequently, pre-operative anemia is incompletely addressed and could be improved using patient blood management programs with engagement and interventions targeted specifically at surgeons [15].

Multiple sources describe an approach to screen and classify pre-operative anemia, and provide guidance on appropriate treatment using algorithms [3, 15, 18, 20–22]. Screening and classification blood work includes a complete blood count (CBC), ferritin, transferrin saturation, folate, vitamin B12, C-reactive protein, and creatinine [18, 20]. Classifications of anemia include iron deficiency anemia, anemia of chronic inflammation, megaloblastic anemia due to folate or vitamin B12 deficiencies, anemia due to renal insufficiency, and other causes of anemia that are beyond the scope of this project [18]. Commonly, anemia in surgical patients is caused by iron deficiency, iron sequestration due to chronic inflammation, or a combination of the two and treatment should involve the administration of oral or intravenous (IV) iron [3, 18, 20]. Oral iron is more cost effective, but can lead to gastro-intestinal (GI) upset and may not be effectively absorbed by the GI tract in patients with iron sequestration and anemia of chronic inflammation [20]. Six weeks of therapy is usually required to see a response as well and this may not be possible in patients undergoing surgery for malignancy as most regulatory bodies mandate shorter wait times to surgery [23]. The use of oral iron is therefore limited, and IV iron should be used in all other cases as it is effective at increasing iron stores, hemoglobin levels, and decreasing the need for perioperative blood transfusions even when administered 2 weeks prior to surgery [3, 15, 18].

Pre-operative anemia may be overlooked due to a lack of awareness of the prevalence and implications, lack of ownership of the problem, lack of resources needed to effectively treat anemia, and a focus on surgery curing the cause of anemia (e.g. bleeding colon cancer) [15, 24]. These issues need to be addressed within the local context to effectively treat pre-operative anemia and improve patient outcomes [15].

Currently, there are an average of 16 patients undergoing major HPB surgery every month at our institution. Pre-operative anemia is present in 39.5% of these patients and only 28.6% of anemic patients are being treated. The aim of this project was to increase the proportion of HPB surgical patients who are treated for pre-operative anemia from 28.6 to 75% by May 31, 2019.

## Methods

### Study design

This was an interrupted time series study which occurred from May 1, 2018 – May 31, 2019.

### Context

This study involved a group of four surgeons who treat benign and malignant conditions involving the liver, pancreas, and biliary system and perform, on average, 16 major surgeries per month in a 1202 bed, tertiary care center. Pre-operative anemia is present in 39.5% of the patient population, and prior to this quality improvement project, only 28.6% of anemic patients were treated with oral or IV iron (baseline data from May–December 2018). Most HPB patients have a relatively short waiting time until surgery (median 27 days) limiting the use of oral iron.

The Surgical Blood Management Program is a local patient blood management program that has existed for many years but has not been leveraged to its full extent by the HPB surgery group. This program is nurse led and provides a full workup and treatment for patients with anemia. Patients receive 200 mg of iron sucrose per infusion and the number of infusions is at the discretion of the Surgical Blood Management Program.

Pre-operative anemia has already been addressed in colorectal surgery patients at our institution. After implementation of a screening and treatment algorithm and leverage of the Surgical Blood Management Program, treatment rates were increased from 20 to 89% (unpublished data). In addition to a high proportion of patients with anemia, the HPB surgeons were already interested in reducing blood loss and blood transfusions intra-operatively. Therefore, a spread initiative was planned using principles from the Institute for Healthcare Improvement (IHI) Framework for Spread [25].

### Improvement diagnostics

To develop a spread plan, we interviewed key stakeholders individually, and then met as a group. The purpose of the interviews was to explain what had been done in the colorectal surgery clinic, and gain feedback on how we could alter and apply the interventions to work in the HPB surgery clinic to ensure a successful spread. The interventions in the colorectal clinic that we focused on included the anemia screening and treatment algorithm, screening blood work, and leverage of the Surgical Blood Management Program. Problems identified with the current process included:

1. The current algorithm is too complicated.
2. It is unclear which blood work tests should be ordered and at what point of time in the patient’s investigation.
3. It is unclear to whom anemic patients should be referred (internal medicine, hematology, or Surgical Blood Management Program).
4. Oral iron is not commonly ordered by surgeons and the optimal formulation and dose needs clarification.

The core change team drilled down to the root cause of each problem using a 5-why’s approach and developed change ideas (Table 1). The team found that the algorithm would be very useful in this spread initiative but required a redesign as there were too many options at the end due to the multifactorial etiology of anemia.

**Table 1:** Problems, root causes, and change ideas identified during the diagnostic phase

Problem	Root Cause	Change Idea
Current algorithm is too complicated	Etiology of anemia can be multifactorial	Re-design the algorithm
Ordering of blood work is unclear	Blood work is required at multiple points in the patient journey	Simplify to CBC and ferritin
Unclear to whom to refer	Multiple programs (medicine, hematology, SBMP)	Designate SBMP as the sole referral destination
Optimal oral iron prescription is unknown	Surgeons do not routinely treat anemia	Create standardized prescriptions

Blood work was being ordered at different times in the patient’s journey and some of this was unnecessary or was repeated by the Surgical Blood Management Program. Additionally, necessary follow up blood work was not ordered at times, delaying care. The nurse lead for the Surgical Blood Management Program recommended that it would be best if the surgeons identified the anemic patients and then left the workup of anemia to the Surgical Blood Management Program. This would reduce the number of times blood work was ordered by the surgeons to one instance, thereby eliminating the need for them to order any follow up blood work and decrease the amount of times patients had blood work drawn.

The previous referral pattern in the HPB clinic was unclear and varied between medicine, hematology, and the Surgical Blood Management Program. The Surgical Blood Management Program was the designated referral destination for anemic patients in the colorectal clinic due to the benefits described above, and it was chosen for the HPB clinic.

Different forms and doses of oral iron are available, and as oral iron is not ordered frequently by surgeons, we made a standardized prescription.

### Interventions

Techniques from the Model for Improvement were used for executing and refining the spread plan. Three of the current interventions used in the colorectal clinic were adapted and applied to the HPB clinic including the use of an anemia screening and treatment algorithm, screening blood work, and leveraging the existing Surgical Blood Management Program. Additionally, standardized oral iron prescriptions were introduced. Plan-do-study-act (PDSA) cycles for all interventions are described in detail in Additional file 1: Appendix A.

#### Pre-operative anemia screening and treatment algorithm

The algorithm was redesigned by the core change team (Additional file 2: Appendix B). Other than identifying anemic patients, providing a prescription for oral iron, or ordering a blood transfusion, we found that all other screening and treatment modalities could be provided by the Surgical Blood Management Program. In order to avoid inappropriate referrals, we included instructions on the algorithm to refer at least 2 weeks prior to surgery to allow for adequate workup and treatment time.

A developmental PDSA was completed by performing a usability test on the two HPB surgeons who were not part of the core change team using simulated patients. Using feedback, we changed the algorithm to better reflect patient flow with regards to when they were first reviewed (and blood work was ordered) and when consent for surgery was signed (and they could be referred to the Surgical Blood Management Program). The initial uptake was good, however, we found that some patients were not being treated and feedback from the four surgeons indicated that they did not always consider treating mildly anemic patients. The core change team discussed this and agreed to treat all anemic patients regardless of severity as recommended by the evidence [15, 20].

#### Blood work

The simplified blood work ordering process was incorporated within the redesigned algorithm (Additional file 2: Appendix B) which can be followed at patient triage (order a CBC and ferritin) and consultation (review the CBC and ferritin).

#### Leverage of surgical blood management program

A referral process was created with the nurse navigator emailing the Surgical Blood Management Program lead. This resulted in high referral rates (90%), and we planned to continue with this process for the project. However, referral rates began to decrease after the second month, and two problems were identified through feedback from the core change team. First, the nurse navigator had too many other responsibilities and could not sustain this process. Second, the nurse navigator took vacation and the nurses who covered were not sending any referrals. We then transferred the referral responsibility (still using email) to the administrative assistant working with the surgeons as they are responsible for booking surgeries and can refer any anemic patient as they are reviewed. This new process has removed the need for the surgeon or nurse navigator to refer during busy clinics and has resulted in an appropriate referral rate of 100%.

#### Standardized Oral Iron prescriptions

Standardized oral iron prescriptions were created and placed this directly on the algorithm with prescribing indications (Additional file 2: Appendix B). We came to this decision using evidence about bioavailability and optimal dosing, as well as which formulation was covered for seniors by the provincial drug plan. A stamp containing the drug information was purchased which could be used by the surgeons to fill out prescriptions. This was placed in the clinic area along with pre-stamped prescriptions and surgeons were made aware of the location.

## Measures

### Outcome measures

Our primary outcome measure was the pre-operative treatment rate of anemia, as defined by WHO criteria, in patients undergoing major HPB surgeries. Our secondary outcome measure was the mean increase in hemoglobin level from initial referral to surgery among anemic patients who have been treated.

### Balancing measure

In order to ensure that there were no adverse patient outcomes, we tracked the number of blood transfusions administered and patients who were referred or treated inappropriately.

### Process measures

Use of the anemia algorithm and ordering of appropriate screening blood work were both tracked by the percentage of patients who were screened for pre-operative anemia with a CBC and referred to the Surgical Blood Management Program. Only patients who were consented for major surgery were included. Referral rates were tracked by the percentage of patients with anemia who were referred. The use of the standardized oral iron prescriptions was tracked by the percentage of patients with anemia correctly receiving a prescription. A process control board was created to track these measures and included patient demographics, hemoglobin and ferritin levels, actions taken, and clinic MD. This was an electronic spreadsheet which could be filled out by the surgeon in clinic or the administrative assistant when patients were booked for surgery. It was updated and emailed on a weekly basis and the data was assessed by the project lead for accuracy with regards to laboratory values and referrals.

All patients who underwent major HPB surgery were included in this quality improvement initiative. Patients were excluded if they did not have surgery (e.g. palliative) or if the surgery was minor (laparoscopic cholecystectomy or diagnostic laparoscopy). Statistical process control charts were used to assess the improvement. P charts were used for classification data. Student’s t-test was used for comparisons between continuous variables and chi-squared test for categorical variables. Squire 2.0 guidelines were used to guide this study [26].

### Ethical considerations

The institutional research ethics board approved this project as a quality improvement initiative.

## Results

Over the course of the study period, 208 patients were included and 39.9% were anemic. See Table 2 for comparisons between the pre and post intervention cohorts.

**Table 2:** Pre and post intervention cohort comparison

	Pre-intervention ***n*** = 124	Post-intervention ***n*** = 84	***p***-value
Anemic patients n (%)	49 (39.5)	34 (40.5)	0.89
Patients screened with CBC and ferritin n (%)	51 (41.1)	54 (64.3)	0.001
Patients referred for treatment of pre-operative anemia n (%)	7 (14.3)	23 (67.6)	< 0.0001
Patients treated for pre-operative anemia n (%)	14 (28.6)	15 (44.1)	0.14
Patients receiving a perioperative blood transfusion n (%)	16 (12.9)	10 (11.9)	0.83
Mean red blood cell units transfused per patient (units)	2	2	0.46
Mean hemoglobin at referral (g/L)	128	128	0.98
Median lead time to surgery (days)	27	29	0.80

There was no statistically significant increase in pre-operative anemia treatment rates from pre-intervention to post-intervention (28.6% vs 44.1%, *p* = 0.14). Although special cause variation was initially seen, this was not sustained. A statistically significant increase in the mean hemoglobin level from 110 g/L to 119 g/L (*p* = 0.03) was seen amongst patients who were treated for pre-operative anemia (*n* = 12). Ferritin levels also increased in these patients from 87 ng/mL to 155 ng/mL (*p* = 0.004).

Our balancing measures revealed that one patient who was not anemic was placed on oral iron and referred inappropriately to the Surgical Blood Management Program. No further treatment was pursued. The overall percentage of patients receiving a perioperative blood transfusion was not changed, no patients received an inappropriate blood transfusion, and the mean number of units transfused per patient was not different (Table 2).

**Fig. 1:**
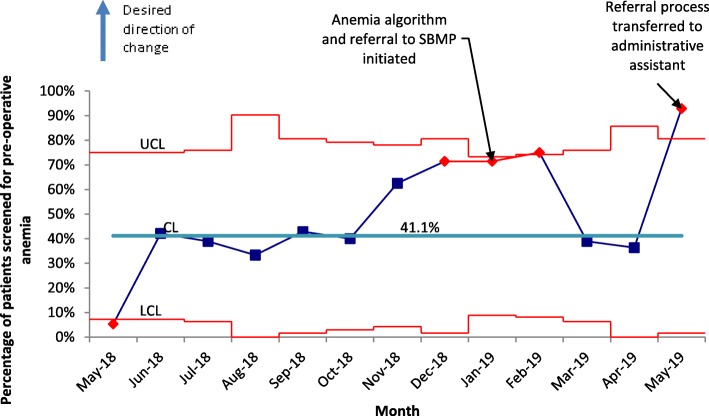
Screening rates for pre-operative anemia in all patients who were consented for major surgery (p-chart). Red markers indicate special cause variation

**Fig. 2:**
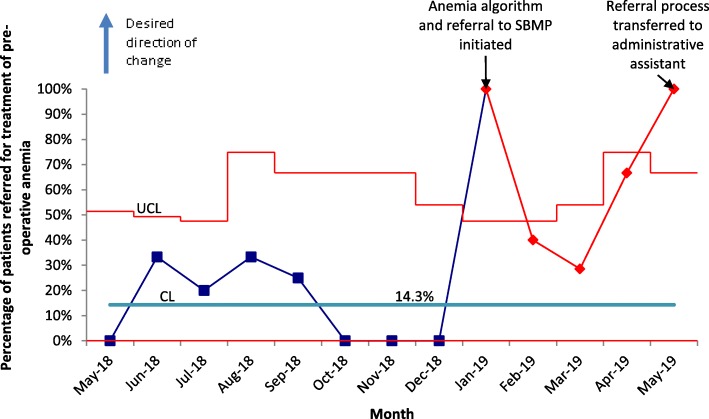
Referral rates for the treatment of pre-operative anemia (p-chart). Red markers indicate special cause variation

Our process measures did reveal an improvement in the screening and referral rates (Table 2, Figs. 1 and 2). Of the 23 anemic patients referred to the Surgical Blood Management Program program post-intervention, 12 were treated. Of the 11 who were not treated, 7 were deemed to be less likely to benefit from treatment, 3 declined treatment, and 1 was referred inappropriately. Oral iron prescriptions were provided to 32% (11/34) of patients.

## Discussion

Over the course of this spread initiative, the pre-operative anemia treatment rate changed from 28.6 to 44.1%. A lot of variation in the treatment rate from month to month was seen and while special cause variation towards an improvement was seen in the first month after implementation, this was not sustained. In patients who did receive treatment for anemia pre-operatively, there was a statistically significant increase in both the hemoglobin and ferritin levels. This did not translate into reduced blood transfusions or mean units of red blood cells per transfused patient. We were able to establish an effective referral process to an existing patient blood management program which has simplified the workup and treatment of anemia for the surgeons.

As the utility of oral iron is limited due to short wait times (29 days) until surgery, and the use of erythropoietin stimulating agents is discouraged due to thrombosis and malignancy concerns, IV iron is often the only choice for the treatment of pre-operative anemia in HPB patients [20]. IV iron and MDCU chair time is a limited resource however, and patients must be triaged based on etiology and severity of anemia. Therefore, referral to the Surgical Blood Management Program did not always translate into a high treatment rate as the available resources had to be allocated to the patients who would likely benefit the most from IV iron. The Surgical Blood Management Program directed most of the resources towards those patients who had a low ferritin level (< 30 ng/mL) with an absolute iron deficiency as opposed to those patients with more of a functional iron deficiency from chronic inflammation. We chose to include all anemic patients as even those with a functional iron deficiency may benefit from IV iron and we would ideally offer treatment to them as well [20]. Work is being done to advocate for more resources to improve access and a recent costing analysis in colorectal patients demonstrated an adjusted increase of hospital costs attributable to anemia of $3027 per case [27]. This is higher when compared to the treatment costs of IV iron (approximately $400 CAD for 2 IV infusions based on local data).

A strength of this study is that it allowed us to pursue a small scale spread from the initial implementation project in colorectal surgery. We were able to learn from this process, especially regarding its sustainability, and make changes in the colorectal clinic as well. As we continue to improve the process within the HPB clinic, we will consider how to spread this even further within the whole department of surgery.

We were limited by the small sample size as we only had an average of 6.4 anemic patients per month. As a result, only a few changes were made to the process during the study period. This affected our sustainability early on and we have taken steps to address this by transferring the referral responsibility to the administrative assistant. We have not yet seen an impact on short or long term clinical outcomes. The number of blood transfusions was not decreased, and this may have been due to the low treatment rate or potentially an inadequate number of IV iron infusions per patient with the mean hemoglobin remaining below normal after treatment. With a longer study period, we will be able to assess other clinical outcomes. This was a spread initiative and the process has been designed to fit the local context. As such, the generalizability of this may be limited, especially if an institution does not have a patient blood management program.

## Conclusions

This study demonstrates a small scale spread focused on the treatment of pre-operative anemia. Although we have not achieved our aim to treat 75% of anemic patients, we have developed an effective referral pathway to an existing patient blood management program and have seen a significant increase in the mean hemoglobin in anemic patients who have been treated pre-operatively. The use of screening and treatment algorithms and patient blood management programs is supported by the literature and the interventions described in this paper can be applied to any surgical patient populations in which pre-operative anemia is present. This should lead to improved patient outcomes by reducing complications, blood transfusions, and length of stay. Future research will be focused on improving the access to IV iron and studying clinical outcomes in patients who are treated pre-operatively over a longer period of time.

## Supplementary information

**Additional file 1: Appendix A.** PDSA cycles.

**Additional file 2: Appendix B.**

## Data Availability

The datasets generated and analyzed during the current study are not publicly available due to privacy reasons.

## Abbreviations

CBC: Complete blood count
HPB: Hepato-pancreato-biliary
IV: Intravenous
MDCU: Medical day care unit
PDSA: Plan-do-study-act
WHO: World health organization
