# Supplementary material for: Improving the treatment of pre-operative anemia in hepato-pancreato-biliary patients: a quality improvement initiative
Source: Patient Saf Surg. 2020 Apr 24;14:18. doi: 10.1186/s13037-020-00239-5 (PMC7181477; doi:10.1186/s13037-020-00239-5)
Supplement: Supplementary file 1 — Additional file 1: Appendix A. PDSA cycles. [file 13037_2020_239_MOESM1_ESM.docx]

**Appendix A**

PDSA cycles

| **Prediction** | **Do** | **Study** | **Action** |
| --- | --- | --- | --- |
| Redesigned anemia algorithm is easy to follow and use | Usability test of redesigned algorithm with 2 HPB surgeons (not part of the core change team) using simulated patients | Feedback suggested that the algorithm is quick and simple to use but does not reflect patient flow exactly and may lead to some confusion around when screening blood work should be ordered | Algorithm modified to separate when the patient is first reviewed (and blood work is ordered) and when they are consented for surgery (and blood work is reviewed) |
| Redesigned anemia algorithm will be used routinely in clinic | Algorithm used for all patients consented for surgery to identify and treat/refer; cycle carried out for two weeks following implementation | Audit of data showed that 67% of patients were screened with a CBC and ferritin for anemia and 100% of anemic patients treated/referred | Continue to monitor use and ensure availability and visibility in clinic area |
| Referral to SBMP will be most reliably completed if done by nurse navigator at the time the patient is consented | Appropriate referrals to the SBMP (sent by email from the nurse navigator) were tracked over four weeks following implementation of the anemia algorithm | Audit of data showed that 90% of patients were referred correctly to the SBMP | Continue to use this process with ongoing monitoring |
| Referral to SBMP by the nurse navigator is not sustainable | Meeting with nurse navigator revealed that this process added too much to current responsibilities and the nurses covering during vacation were not sending referrals | Audit of data from process control board showed that referrals to the SBMP had declined to 27.5% of eligible patients by the month of March, 2019 | Discuss transfer of referral responsibility to administrative assistant with core change group |
| Referral to SBMP will be sustained long term if responsibility is transferred to an administrative assistant | Assessment of appropriate referrals to SBMP over two weeks | Audit of data from process control board and SBMP showed that 100% of patients were referred correctly to the SBMP | Continue to use this more sustainable referral process with ongoing monitoring |
| Standardized prescriptions will simplify the ordering of oral iron | Audit of correct ordering of oral iron over two weeks | Only one patient required oral iron and was provided with a prescription; feedback suggested having pre-stamped prescriptions available | Continue to monitor and provide pre-stamped prescriptions |
