# Supplementary material for: Improving the treatment of pre-operative anemia in hepato-pancreato-biliary patients: a quality improvement initiative
Source: Patient Saf Surg. 2020 Apr 24;14:18. doi: 10.1186/s13037-020-00239-5 (PMC7181477; doi:10.1186/s13037-020-00239-5)
Supplement: Supplementary file 2 — Additional file 2: Appendix B. [file 13037_2020_239_MOESM2_ESM.docx]

**Appendix B**

Figure 1 – Previous anemia screening and treatment algorithm


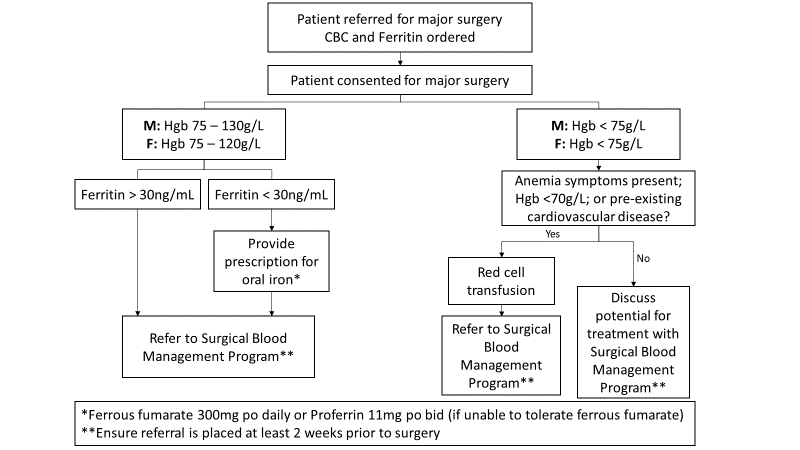


Figure 2 – Redesigned anemia screening and treatment algorithm
